# What Is Asthma?

**DOI:** 10.3390/jcm10061282

**Published:** 2021-03-19

**Authors:** Luis García-Marcos

**Affiliations:** 1 Paediatric Allergy and Pulmonology Units, ‘Virgen de la Arrixaca’ University Children’s Hospital, University of Murcia, 30100 Murcia, Spain; lgmarcos@um.es; 2 Biomedical Research Institute of Murcia (IMIB-Arrixaca), 30120 Murcia, Spain; 3 Network of Asthma and Adverse and Allergic Reactions (ARADyAL), 28029 Madrid, Spain

Asthma is…. what? A symptom, a condition, a disease? I have been speaking about asthma and I have been hearing about asthma for the last 40 years. I have heard about allergy and about phenotypes in children and in adults; I have also heard of endotypes and even of “other-types”. I come from the time that having a space mask was a privilege and now I have to decide what biological therapy is better for a specific child and to “tailor” a treatment following the guidelines of the Lancet commission [1]. Things seem to have changed very much, but, in essence, asthma remains a mystery. Maybe because, as Fernando Martínez reminded us some time ago, asthma is now like fever was in the late 19th century, which was considered a disease [2].

Thus, it is also a good idea to dedicate some discussion to asthma and the new achievements related to the disease, but also to the old paradigms of this condition… or should I have said symptom? The *Journal of Clinical Medicine* had the good idea of launching a Special Issue on asthma, but the not so good one of asking me to organize it. Anyhow, good friends (“that’s what friends are for”, as Dionne Warwick put it) decided to accompany me in this task and contribute with their knowledge.

John Warner decided to revisit an old but never solved question about the commencement of humanity—the egg or the chicken [3]—and, in masterful fashion, he led us from the basics to personalized treatment, taking into account the so-called “allergic march”. Andy Bush, always on the cutting edge of knowledge, explains to us what child is a good candidate for biological therapy and revisits the need to establish phenotypes (which maybe not be as stable as we would like them) to make better decisions [4]. Phenotypes (in adults) are also the topic of the paper by Sara Maio and the AGAVE Pisa group [5]. They very elegantly show, through latent transition analysis, that some environmental factors are crucial for defining some phenotypes and even that there may be specific forms of asthma associated to tobacco smoke or air pollution.

Alan Kaplan and colleagues [6] suggest, with all of my support, that it is time to shift to SABA-ICS reliever medication, and I would also add in Paediatrics. In this same department of general treatment, Carlo Caffarelli and colleagues [7] update the usage of sublingual immunotherapy in children. This somewhat different approach to immunotherapy for asthma has still some detractors, but seems to be becoming adept as evidence accumulates that it is a good option when the right patient is chosen… or perhaps I should put it the other way around: when the patient meets the criteria for its indication.

My good friend and colleague Manuel Sánchez-Solis continues the saga of lung development in the early stages and later asthma with new data on children born prematurely [8] and elegantly shows that a limited lung function during infancy is a risk of future wheezing and of severe wheezing episodes. I think we forget about lung maturation when dealing with asthma and it is quite probable that, in a totally separate path of the TH2 one, ill-matured lungs are much more prone to asthma symptoms in childhood and even later on than correctly matured lungs. Lung development and maturing is probably the reason why preterm birth is a risk for asthma and, as showed by Vogt et al., every week (of gestation) counts [9].

In addition, what about the markers? We desperately need markers of asthma and of asthma phenotypes. Especially, early markers of the condition which might point to primary prevention measures. After the tailored treatments this is, in my opinion, the way we should go if we want to really diminish the burden of asthma. In this Special Issue, Giulia Scioscia and her Italian colleagues [10] add another brick to the wall and show that estradiol in airways may well be a good marker of postmenopausal severe asthma and help to phenotype severe asthmatic patients with neutrophil inflammation.

In the epidemiology arena, Carmen Frontela and colleagues [11] indirectly put the focus on another forgotten road to asthma that has not been sufficiently insisted on: oxidative stress. Yes, it is not a pathway as explored and consistent as the Th2 one, but there is already enough evidence that mechanisms related to the handling of free radicals have a role in asthma [12]. However, there is also obesity and its double adverse effects on lungs: the low-level inflammation and the usually forgotten relationship with the lack of enough exercise and the need to stretch bronchial muscles to have then well developed and “in good shape” in order to avoid over-contraction [13]. Did I mention that this is part of the Western lifestyle package?

This Special Issue has also the privilege and the honor of including the first results from the Global Asthma Network (GAN) Phase I study [14]. Its predecessor, the International Study of Asthma and Allergies in Childhood (ISAAC), made possible in great part by some of the authors of this paper, namely, Innes Asher, Philippa Ellwood, Neil Pearce and David Strachan, was an enormous breakthrough in asthma epidemiology in children. GAN also includes the adult population. I am sure this paper is the starting gun of a new story which promises to be fascinating.

This Special Issue is probably not going to solve the problem, but will be interesting to read as it contains valuable information of almost every part of the asthma spectrum. It is the job of the reader to try and put the pieces in the right place (if there is a right place) to try and get a world envisioned by the Global Asthma Network where “no-one suffers from asthma”.

Oops… also, as warned by the Greek friends [15], please do not confound asthma with bronchiectasis… or have some patients with bronchiectasis symptoms of asthma?... So, then… what is asthma?
